# *Mycobacterium bovis* in a European bison (*Bison bonasus*) raises concerns about tuberculosis in Brazilian captive wildlife populations: a case report

**DOI:** 10.1186/s13104-017-2413-3

**Published:** 2017-02-10

**Authors:** Cristina Kraemer Zimpel, Juliana Sperotto Brum, Antônio Francisco de Souza Filho, Alexander Welker Biondo, João Henrique Perotta, Cristina Corsi Dib, Marcelo Bonat, José Soares Ferreira Neto, Paulo Eduardo Brandão, Marcos Bryan Heinemann, Ana Marcia Sa Guimaraes

**Affiliations:** 1 Departamento de Medicina Veterinária Preventiva e Saúde Animal, Faculdade de Medicina Veterinária e Zootecnia, Universidade de São Paulo, Av. Prof. Dr. Orlando Marques de Paiva, 87-Cidade Universitária, São Paulo, SP CEP 05508-270 Brazil; 2 Departmento de Medicina Veterinária, Universidade Federal do Paraná, Rua dos Funcionários, 1540, Curitiba, PR CEP 80035-060 Brazil; 3 Instituto Biológico de São Paulo, Av. Conselheiro Rodrigues Alves, 1252, São Paulo, SP CEP 04014-002 Brazil; 4 Departamento de Pesquisa e Conservação da Fauna-Zoológico Municipal de Curitiba, Rua João Miqueletto, s/n, Curitiba, PR CEP 81860-270 Brazil; 5 Department of Microbiology, Institute of Biomedical Sciences, University of Sao Paulo, 1374 Prof. Lineu Prestes Avenue, Cidade Universitária, São Paulo, SP CEP 05508-900 Brazil

**Keywords:** *Mycobacterium bovis*, Tuberculosis, Bison, Captive animals, Zoo

## Abstract

**Background:**

Tuberculosis caused by *Mycobacterium bovis* is an important worldwide zoonosis and has been reported to cause clinical disease in several animal species, including captive wildlife. This report describes a case of *M. bovis* infection in a European bison from a Brazilian zoo and compiles a number of literature reports that raise concern regarding tuberculosis among captive wildlife in Brazil.

**Case presentation:**

A 13 year-old captive-born male bison (*Bison bonasus*) from a Brazilian zoo began presenting weight loss, diarrhea and respiratory symptoms, which inevitably led to his death. At the animal’s necropsy, inspection of the thoracic and abdominal cavities revealed multiple enlarged lymph nodes, ranging from 4 to 10 cm, and pulmonary nodules containing caseous masses with firm white materials consistent with mineralization. Histopathology findings showed a significant amount of acid-alcohol resistant bacilli compatible with *Mycobacterium* spp. Specimens from lymph nodes and lungs were cultured on Petragnani and Stonebrink media, and specific PCR assays of the bacterial isolate identified it as *M. bovis*.

**Conclusion:**

The European bison reported herein died from a severe form of disseminated tuberculosis caused by *M. bovis*. A review of the available literature indicates possible widespread occurrence of clinical disease caused by *M. bovis* or *M. tuberculosis* affecting multiple animal species in Brazilian wildlife-related institutions. These likely underestimated numbers raise concern regarding the control of the disease in captive animal populations from Brazil.

## Background

Tuberculosis is an important zoonosis caused by microorganisms of the *Mycobacterium tuberculosis* complex (MTC) and can affect human beings and several other animal species. *Mycobacterium bovis* and *Mycobacterium tuberculosis* are the most important pathogens of this complex that can cause disease in wildlife [1, 2]. Tuberculosis in zoo specimens or free-ranging populations located in national parks constitutes the majority of the reported cases of the disease in the literature [1]. Captive or movement-restrictive conditions and proximity with infected humans or livestock may contribute to the disease occurrence in these populations [3].

Over the years, many reports have detected clinical tuberculosis caused by *M. bovis* or *M. tuberculosis* in captive animals from Brazilian zoos [4–10]. Given the lack of standardized treatment protocols, these animals are frequently euthanized to avoid further spread of the disease. Besides representing a public health risk, the presence of tuberculosis in captive animals, as well as free-ranging species, represents a major negative impact on animal conservation [1]. Herein, we describe a case of severe disseminated disease caused by *M. bovis* in a European bison from a Brazilian zoo and compile a number of literature reports that raise concern regarding tuberculosis among captive wildlife in Brazil.

## Case presentation

**Fig. 1:**
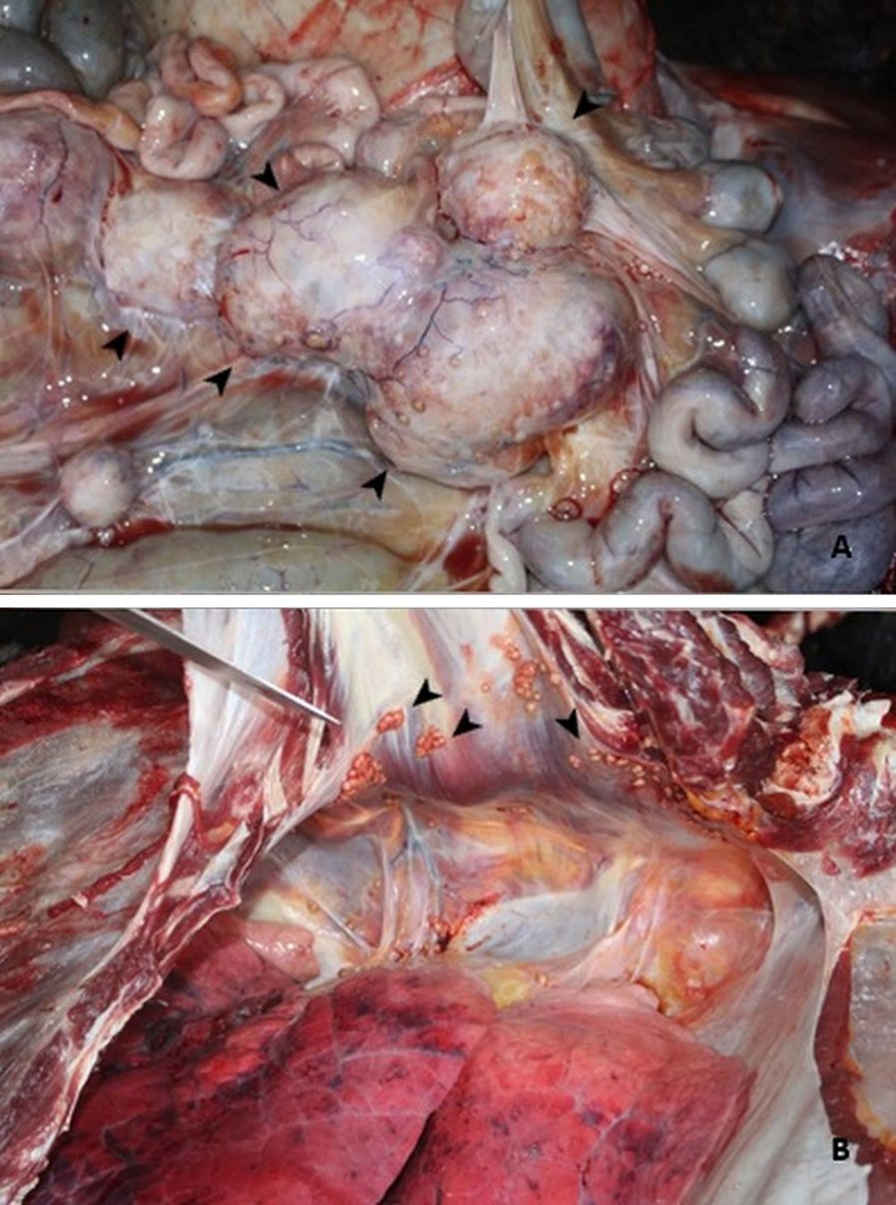
Necropsy findings of *Mycobacterium bovis* infection in a captive European bison (*Bison bonasus*). **A** Abdominal cavity demonstrating mesenteric lymph nodes ranging from 4 to 8 cm (*black arrowheads*). **B** Thoracic cavity containing multifocal to confluent granulomas (*black arrowheads*)

As of 2013, the Curitiba Zoological Garden, located in the Southern State of Paraná, Brazil, held 176 mammals, 480 birds, and 1101 reptiles, from 132 different species. In the second semester of that year, a 13 year-old captive-born male bison (*Bison bonasus*) began presenting weight loss, diarrhea, coughing and purulent nasal secretion that inevitably led to his death. At this time, the animal was kept in a wire-fenced enclosure solely with his mother. This particular enclosure is located at the border of the zoological garden, which is partly surrounded by an environmentally protected area of a river basin. At the animal’s necropsy, a poor body condition score and pale mucous membranes were noted. In the abdominal cavity, mesenteric lymph nodes were enlarged and measured from 4 to 8 cm in diameter (Fig. 1A). The iliac lymph node was 8 cm in diameter, and was causing a hydroureter as it pressed the right ureter against the abdominal wall. A hemolymph node, measuring 3.5 cm in diameter, was observed in the costal surface of the abdominal wall. The posterior ventral and right costal surfaces of the abdominal wall contained multifocal to confluent, yellow–orangish areas of consolidation, 1 cm in diameter. In the thoracic cavity, mediastinal lymph nodes (cranial, tracheobronchial, caudal) were enlarged, measuring from 7 to 10 cm in diameter. The right cranial lung lobe showed thickening of the pleura and fibrin deposition, characteristic of pleuritis, as well as small hemorrhagic foci. Multifocal to confluent, yellow–orangish areas of consolidation, from 1 to 4 cm in diameter, were observed in the lungs, predominantly on the right side (Fig. 1B). All these enlarged nodes were mostly firm and pale yellow in appearance, with a central caseous mass containing firm white material consistent with mineralization.

**Fig. 2:**
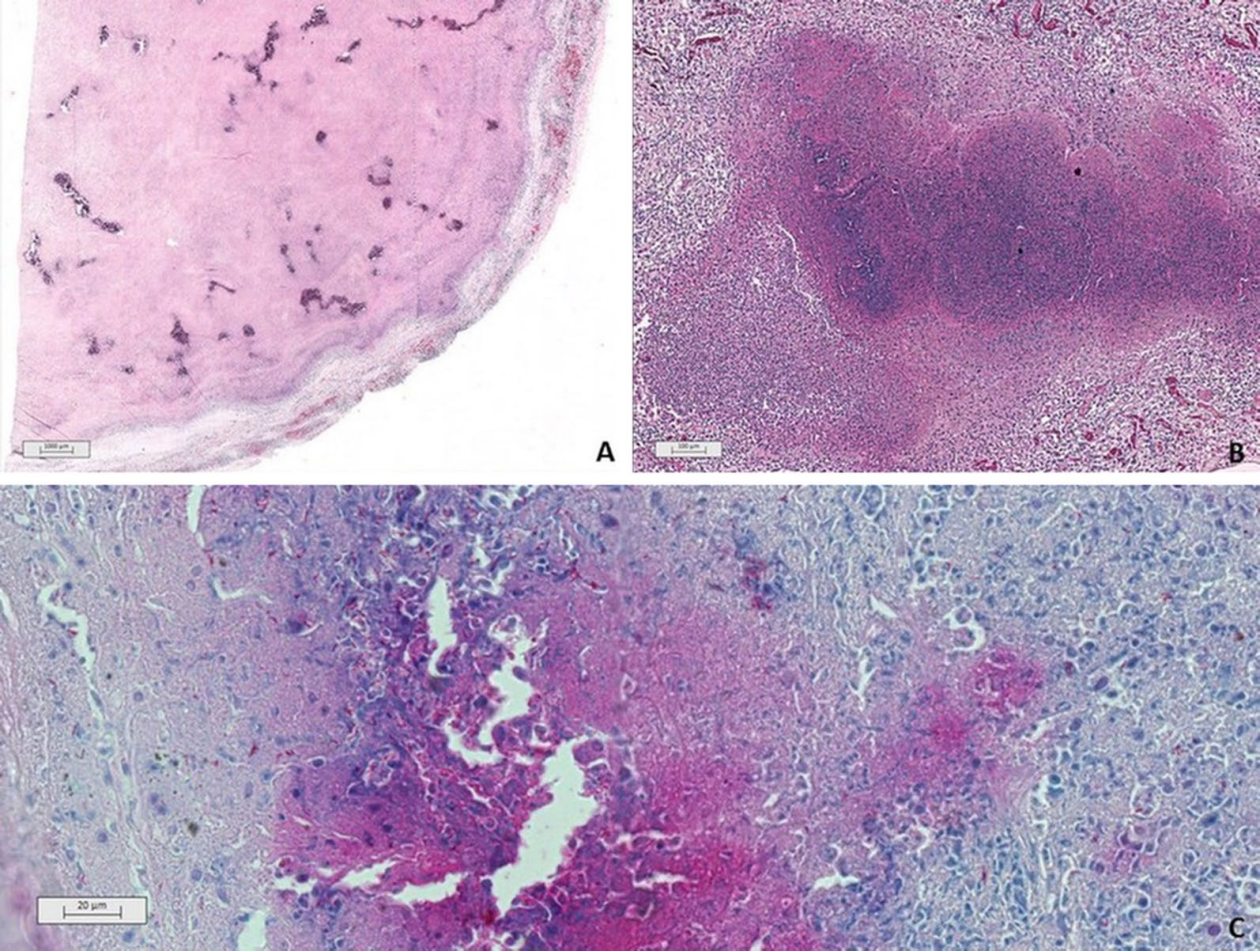
Histopathology of tissue samples collected from a captive European bison (*Bison bonasus*) infected with *Mycobacterium bovis.* **A** Mesenteric lymph node showing multiple and confluent granulomatous inflammation with caseous necrosis and central mineralization pushing the lymph node tissues against its capsule. Haematoxylin and eosin stain. **B** Lung tissue with foci of granulomatous inflammation characterized by central necrosis surrounded by epithelioid macrophages. Lymphocytes, plasmocytes and occasionally well-differentiated fibroblasts were observed delimiting the granuloma. Haematoxylin and eosin stain. **C** Histochemistry of a section from the lung granuloma showing a great number of acid-alcohol resistant -alcohol resistant bacilli (BAAR) consistent with *Mycobacterium* spp. Ziehl–Neelsen stain

Samples from the mesenteric lymph node, pleura, and lungs were collected for histopathology. Lymph nodes and pleura sections showed similar granulomas with a high quantity of a central amorphous, basophilic material suggestive of mineralization. Lymph node tissues have been displaced against the capsule by the granulomas (Fig. 2A). Lung sections showed multifocal to confluent granulomas consisting of central necrosis surrounded by epithelioid macrophages and a few multinucleated giant cells. Lymphocytes, plasmocytes and occasionally well-differentiated fibroblasts were surrounding the granuloma (Fig. 2B). A tentative tuberculosis diagnosis was made on the basis of the clinical pathological findings and due to previous reports of MTC infecting peccaries (*Tayassu tajacu*) [4], a tapir (*Tapirus terrestris*) [6], an aoudad (*Ammotragus lervia*) and a waterbuck (*Kobus ellipsiprymnus*) (unpublished data) from the same zoo. Therefore, Ziehl–Neelsen staining was performed in a section of the lung granuloma and the presence of acid-alcohol resistant bacilli (BAAR) consistent with *Mycobacterium* spp (Fig. 2C) was identified.

**Fig. 3:**
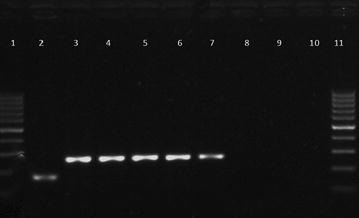
Gel electrophoresis of PCR products for identification of *Mycobacterium bovis*, using primers RD 4 [13]. *Lanes 1* and *11*: 100 bp ladder; *2*: *M. tuberculosis* H37Rv, 172 bp; *3*: *M. bovis* strain AN5, 278 bp; *4*–*7*: DNA extracted from colonies isolated in Stonebrink media; *8* and *9*: empty wells; *10*: negative control (water)

To identify the bacterial species, specimens from lymph nodes and lungs were treated by Petroff method and cultured on Petragnani and Stonebrink media at 30 and 37 °C for 90 days and inspected weekly for bacterial growth. Eugonic and dysgonic colonies suggestive of mycobacteria growth were then propagated in Stonebrink media. DNA from these cultures was extracted by a boiling method [11] and bacterial species identification was performed using PCR protocols [12, 13]. All colonies were molecularly identified as *M. bovis* (Fig. 3).

## Discussion

Captive European bison are exotic and rare in Brazil. Inbreeding may be a common practice to increase the number of specimens. Therefore, the bison described herein was inbred, being an offspring of a female bison with her father. Briefly, the first bison couple to be kept in the Curitiba Zoological Garden originated from another zoo located 400 km away and they were also born in captivity. They were transferred to Curitiba in the 1990s, and soon thereafter had an offspring of female twins. In 1999–2000, the father copulated with one of his daughters and generated the bison presented as a case in this report. According to the zoo archives, the first, original couple died in the early 2000s from reasons unrelated to tuberculosis. The aunt of the bison described herein died in 2008, whereas, the bison’s mother died in 2015, 2 years after him. Also between 2009 and 2010, two free-ranging coatis (*Nasua nasua*) from the local fauna were found dead surrounding the bison’s enclosure. Necropsy findings of the aunt, the mother, and both coatis were compatible with tuberculosis, showing granulomatous lesions in various organs and a number of acid-alcohol resistant bacilli by Ziehl–Neelsen staining. Unfortunately, tissue samples were not available for culture or molecular testing and the source of infection for all cases remains unknown.

With the current information, it is not possible to determine if the local free-ranging coati population may have served as the original infection source of this possible outbreak in the bison or vice versa. In both occasions (the aunt’s death in 2008 and the bison’s death in 2013), bison that were still in the enclosure were subjected to a preventive oral isoniazid, rifampicin and pyrazinamide treatment for eight months, even in the absence of clinical signs. Voluntary acceptance of medicated food by the animals was an issue, according to zoo records. It is also important to mention that this enclosure is isolated and does not allow contact between animals and visitors. Farm implements, uniforms and boots were exclusive of the bison’s enclosure to prevent possible disease spread within the zoo. All zookeepers and veterinarians used personal protective equipment and underwent additional tuberculin skin test and chest radiographs in each occasion (once-a-year mandatory practice in the zoo). Fortunately, zoo employees have never shown positive results for tuberculosis.

In 2001, a National Program of Control and Eradication of Bovine and Buffalo Brucellosis and Tuberculosis (PNCEBT) [14] was created in Brazil aiming to reduce the frequency of both diseases in commercial bovine herds. Wild animals, unfortunately, were not considered in the PNCEBT. Also, Brazil reports 70,000 cases and 4000 human deaths for *M. tuberculosis* yearly, and potential spillover of *M. tuberculosis* to zoo animals has been reported [6]. Since tuberculosis in wild animals is not a notifiable disease in the country, cases have been sporadically and scarcely documented. In addition to the case described herein, *M. bovis* or *M. tuberculosis* infections in wild animals have been reported in at least nine Brazilian zoos or wildlife-related institutions [4–10]. In particular, a more comprehensive study has described the isolation of *M. tuberculosis* or *M. bovis* from samples of captive llama (*Lama glama*), capybara (*Hydrochaeris hydrochaeris*), elephant (*Elephas maximus*), parrot (*Amazona aestiva*) and tufted capuchin (*Cebus* sp.) from four Brazilian institutions [10]. The actual extent of tuberculosis in Brazilian captive populations is therefore uncertain and likely to be underestimated.

The above-mentioned tuberculosis reports from zoos do not represent comprehensive prevalence and incidence data related to the disease in Brazilian captive wildlife. It is only possible to conclude that at least nine different zoos have reported clinical disease due to MTC in their animals over the past 8 years. Considering the negative impact tuberculosis has on public and animal health (e.g. risk of zoonotic transmission, possible euthanasia of endangered species), the authors believe that tuberculosis in zoo populations should be seriously taken into consideration when designing novel public and animal health actions related to the disease in the future.

One point to be addressed in further management plans and studies, for example, is the fact that Brazilian zoos often trade specimens and/or receive animals with unknown disease status from illegal trafficking confiscation or from other situations that preclude their reintroduction into the wild. Accurate diagnostics is pivotal to any infectious disease management. The absence of standardized diagnostic protocols for tuberculosis in several animal species may allow infected specimens to be easily introduced into captive populations, facilitating the disease spread and maintenance within and among institutions. Despite the development of tuberculosis diagnostic tests and control guidelines for elephants, non-human primates and non-domestic hoofstock [15–17], a pan-host diagnostic test is still lacking. The traditional tuberculin skin test is not applicable to all animal species and may present variable sensitivity and specificity, not identifying all truly infected animals or showing false positives due to animal exposure to environmental mycobacteria [18, 19]. Therefore, the development of sensitive and specific diagnostic tests and the establishment of specific wildlife legislation regulating animal trade, disease detection and reporting may aid in the control of tuberculosis in zoo animals.

A second point to be addressed is the identification of epidemiological factors related to tuberculosis spread inside zoos (e.g. *Mycobacterium* spp. environmental persistence and animal shedding routes [20, 21], aerosol transmission among different enclosures, contamination of materials and workers caring for animals, among others). Tuberculosis is a chronic disease and cases may occur over a long period of time. It is necessary to keep accurate animal records and to pursue molecular genotyping of *M. bovis* or *M. tuberculosis* isolates in each case. With such information, it is possible to detect spillovers from livestock or humans to wildlife, clonal outbreaks, and/or if genotypes brought with exotic species from abroad are being perpetuated within Brazilian zoos.

## Conclusion

The European bison reported herein died from a severe form of disseminated tuberculosis caused by *M. bovis*. A review of the zoo archives indicates a possible outbreak involving local free-ranging coatis and other bison from the same enclosure. Also, available literature reports indicate widespread occurrence of clinical disease caused by *M. bovis* or *M. tuberculosis* affecting multiple animal species in Brazilian wildlife-related institutions. The control of the disease has been hampered by the unavailability of efficient and fast diagnostic tests and the lack of legislation regarding the control of tuberculosis in wild animal species. The occurrence of MTC in these animal populations represents a risk to animal conservation and public health in Brazil.

## Abbreviations

BAAR: acid-alcohol resistant bacilli
MTC: *Mycobacterium tuberculosis* Complex
PNCEBT: National Program of Control and Eradication of Bovine and Buffalo Brucellosis and Tuberculosis
